# Recent Developments and Prospects of M13- Bacteriophage Based Piezoelectric Energy Harvesting Devices

**DOI:** 10.3390/nano10010093

**Published:** 2020-01-02

**Authors:** In Woo Park, Kyung Won Kim, Yunhwa Hong, Hyun Ji Yoon, Yonghun Lee, Dham Gwak, Kwang Heo

**Affiliations:** Department of Nanotechnology and Advanced Materials Engineering, Sejong University, Seoul 05006, Korea; lovepiw@naver.com (I.W.P.); saheon7@gmail.com (K.W.K.); monkey1434@gmail.com (Y.H.); hyunji9193@naver.com (H.J.Y.); alwayscoast@gmail.com (Y.L.); whalephant13@gmail.com (D.G.)

**Keywords:** virus, M13 bacteriophage, energy generator, piezoelectric, self-assembly, genetic engineering

## Abstract

Recently, biocompatible energy harvesting devices have received a great deal of attention for biomedical applications. Among various biomaterials, viruses are expected to be very promising biomaterials for the fabrication of functional devices due to their unique characteristics. While other natural biomaterials have limitations in mass-production, low piezoelectric properties, and surface modification, M13 bacteriophages (phages), which is one type of virus, are likely to overcome these issues with their mass-amplification, self-assembled structure, and genetic modification. Based on these advantages, many researchers have started to develop virus-based energy harvesting devices exhibiting superior properties to previous biomaterial-based devices. To enhance the power of these devices, researchers have tried to modify the surface properties of M13 phages, form biomimetic hierarchical structures, control the dipole alignments, and more. These methods for fabricating virus-based energy harvesting devices can form a powerful strategy to develop high-performance biocompatible energy devices for a wide range of practical applications in the future. In this review, we discuss all these issues in detail.

## 1. Introduction

Recently, energy harvesting from biomechanical movement has attracted a great deal of interest in wearable, sustainable, and biomedical technologies [1,2,3]. Especially, piezoelectric devices have been actively studied for bio-implantable application, because they can easily generate energy by using simple motions and vibrations without any other external source [4,5,6]. General piezoelectric materials have the ability to generate electrical charges from applied mechanical stress. Although the energy output from piezoelectric generators may not be as large as that from other alternative energy sources, these also have their own advantages, such as simple device structures and various material groups.

After Jacques and Pierre Curie discovered piezoelectricity from quartz in 1880, various piezoelectric materials were developed from ceramics to natural biomaterials. Among them, ceramic materials such as lithium niobate (LiNbO_3_) [7], potassium niobate (KNbO_3_) [7], lithium tantalate (LiTaO_3_) [8], barium titanate (BaTiO_3_) [9], lead zirconate titanate (Pb[Zr_x_Ti_1−x_]O_3_) [10], and etc., were most investigated due to their superior piezoelectric properties. Lead zirconate titanate (PZT) has become the most common piezoelectric material in practical application today. However, as the issue of toxicity in lead-containing devices is starting to appear, extensive study has been conducted to replace it with lead-free piezoelectric materials [11]. For this purpose, several ceramics (bismuth ferrite (BiFeO_3_), sodium niobate (NaNbO_3_), barium titanate (BaTiO_3_), bismuth titanate (Bi_4_Ti_3_O_12_), quartz, etc.) [12,13,14] and organic materials (polyvinylidene fluoride (PVDF), polyvinylidene chloride (PVDC), etc.) [15,16] were found. Particularly, single-crystal zinc oxide (ZnO) nanostructures with wurtzite structure exhibit larger piezoelectric constants than those of bulk ZnO. However, the poor biocompatibility and brittle characteristics of these piezoelectric materials limit their applications in wearable and biomedical applications [17].

Otherwise, biomaterials have been regarded as promising alternative materials due to their good biocompatibility, non-toxicity, and environmental friendliness. After the first discovery of the piezoelectric effect of bone, the piezoelectric properties of diverse natural biomaterials such as wood, bone, hair, dentin, tendon, and collage were investigated [18,19,20,21,22,23]. Since these natural materials exhibit very weak piezoelectricity and are difficult to mass-produce, there is a limit to use for practical applications. Recently, there has been a growing interest in the biopiezoelectric materials to overcome these limitations. Polysaccharide materials [24,25,26,27], viruses [28,29,30,31], and self-assembled biomaterials [32,33] have been identified as good candidates, because their piezoelectric constants are higher than those of previous natural biomaterials, and they are possible to mass-produce. Especially, it has been found their physical and chemical properties can be modulated by their morphology, surface charges, and phases, and the piezoelectric response is directly related to these. Furthermore, the discovery of piezoelectricity in bone [34,35,36], which has been the most frequently studied tissue, aroused great interest because it seemed to provide an important key to understanding bone physiology. Researchers hypothesized that bone’s piezoelectric signal by physical stimulation could regulate bone growth, repair, wound healing, and tissue regeneration [37,38,39]. In addition, piezoelectric biomaterials also have several advantages for use in sensors [40], energy storage [41], energy harvesting, and other areas [42]. Despite these advantages of biopiezoelectric materials and their potential applications, a comprehensive review of virus-based piezoelectric energy harvesting devices have not been reported.

In this short review, we provide an overview of M13 bacteriophages (phages) as superior biopiezoelectric materials for piezoelectric energy harvesting applications. In addition, we discuss in detail the piezoelectric properties of M13 phages and the fabrication of M13 phage-based piezoelectric energy harvesting devices. It is expected that this review will inspire the design of novel biomaterials and the development of functional devices for energy harvesting, sensing, biomedical applications, and other applications.

## 2. Biological Building Block for Piezoelectric Energy Harvesting Devices: M13 Bacteriophages

Due to their unique structural, biological, and physical properties, the M13 phage is the most attractive candidate in biomaterials for mimicking natural structures and developing novel piezoelectric energy harvesting devices. Especially, from the engineering point of view, M13 phages have several advantages, such as (1) structural similarity with collagens; (2) mass-producibility by bacteria infection and mass-amplification; (3) surface tunability through genetic engineering; (4) possibility of forming a highly-ordered structure via self-assembly; and (5) superior piezoelectric properties.

**Figure 1:**
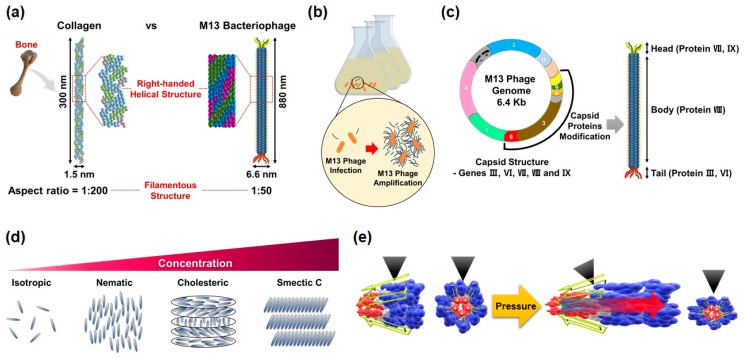
Schematic diagram showing the main characteristics of M13 bacteriophages (phages). (**a**) Structural similarity between natural collagen and M13 phages. Both biomaterials are filamentous structures with high aspect ratio and have right-handed helical structures. (**b**) Infection and mass-amplification of M13 phages. A huge amount of phages can be produced in infected bacteria by using the metabolic reactions of the host cell. (**c**) M13mp phage vector and surface modification through genetic engineering. We can design the molecular structures of the phage’s outer surfaces in accordance with the desired properties and display the related peptide motif on the coat proteins of M13 phages. (**d**) Liquid crystalline phase transition of M13 phages. M13 phages exhibit a lyotropic liquid crystalline phase transition due to their helical structure, nanofibrous shape, monodispersity, and functional motifs. (**e**) Piezoelectric properties of M13 phages, which enable us to make piezoelectric energy harvesting devices.

The M13 phage is a filamentous bacteriophage composed of circular single-stranded deoxyribonucleic acid (ssDNA) and capsid proteins. ssDNA is encapsulated in approximately 2700 copies of the helically arranged major coat protein pVIII, and five to seven copies of two different minor coat proteins (pIX, pVI, pIII, pVII) on the ends (Figure 1a). The diameter and length of M13 phages are about 6.6 nm and 880 nm, respectively [43]. Since the structural characteristic of phages is very similar to the structure of the human collagen, the M13 phages are quite capable of mimicking nature’s hierarchical structures based on collagen [44]. These M13 phages, which are perfectly identical copies, can be mass-produced using the living characteristic of viruses. M13 phages are any group of viruses which carry out a lysogenic infection in which the phage inserts its genome into the bacterial genome. The minor coat protein pIII attaches to the receptor of the host bacteria and infects the bacteria. A huge amount of phages can be produced in an infected bacteria using the metabolic reactions of the host cell (Figure 1b). The infected cells are not involved in the cell lysis, but a decrease in the rate of cell growth [45].

Recent advances in genetic engineering make it possible to modulate the peptide sequence of phage proteins as desired. By using the recombinant DNA technique and M13mp phage vectors, we can design the molecular structures of surfaces according to the required properties and easily display the related peptide motif on the coat proteins of M13 phages (Figure 1c). This ability of M13 phages is a unique feature that distinguishes them from other nano and biomaterials [45].

In addition, M13 phages exhibit a lyotropic liquid crystalline phase due to their helical structure, nanofibrous shape, monodispersity, and expressed functional motifs (Figure 1d). According to the concentration of the phage suspension, the resulting structures of M13 phage films change from an isotropic phase to a cholesteric phase in a controlled manner [43]. Owing to these characteristics, we can prepare highly-ordered crystalline structures in a large area, which allows us to fabricate functional devices.

Lastly, recent studies have shown that M13 phages have excellent piezoelectric properties, which are larger than other natural biomaterials [28]. This makes it possible to fabricate high-performance piezoelectric energy harvesting devices (Figure 1e).

## 3. Introduction to Piezoelectric Effect

**Figure 2:**
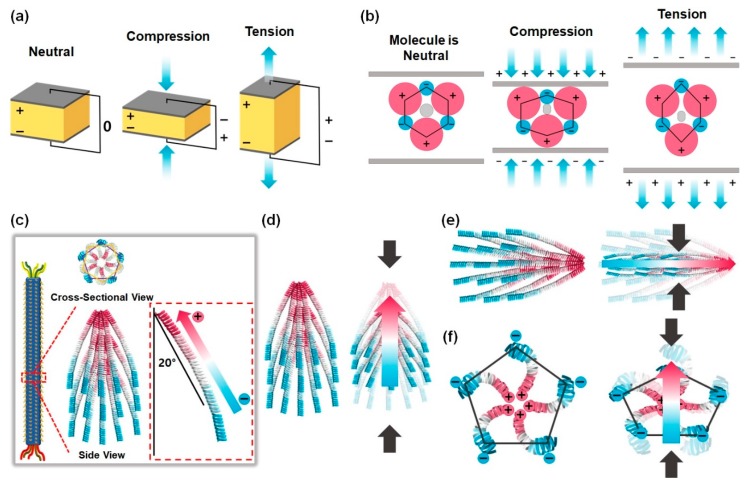
Schematic diagram showing the working mechanism of the piezoelectric effect. (**a**) Diagrammatic demonstration of the piezoelectric effect. (**b**) Schematic illustration showing the working mechanism of the piezoelectric effect in crystal structures. In equilibrium, the charges of the unit cell are neutral (no net dipole moment). When mechanical stresses are applied, a net dipole moment and electrical polarization arise in piezoelectric materials. (**c**) Schematic diagram showing structural characteristics of M13 bacteriophages. M13 phages have five-fold rotational symmetry, two-fold screw symmetry, and no inversion center. Schematic illustrations showing the working mechanism of the piezoelectric effect based on M13 phages when the stress is applied along the phage long axis (**d**) and phage short axis (**e**,**f**).

Piezoelectricity is a phenomenon of coupling between the electrical and mechanical states of a material by crystal deformation. When piezoelectric materials are mechanically stressed and deformed, the positive and negative charge centers shift in the materials, which then results in an external electrical field and a current flow. The opposite can also happen. When an electrical field is applied to the materials, the piezoelectric materials are stretched or compressed (Figure 2a).

This direct piezoelectric effect was first discovered in 1880 by Paul-Jacques Curie, Pierre, and Marie Curie. They combined the knowledge of pyroelectricity with their understanding of crystal structures and behavior, and demonstrated the first piezoelectric effect by using crystals of quartz and Rochelle salt. Since then, many researchers have discovered and reported the piezoelectric properties of organic [15,16], inorganic [7,8,9,10,11,12,13,14,17], and biomaterials [18,19,20,21,22,23,24,25,26,27,28,29,30,31,32,33].

These piezoelectric characteristics originate from the deformation of the crystal structure and charge rearrangement within the material. In the equilibrium state, the arrangement of charges within the material lattice is neutral. However, there will be a charge redistribution within the unit cell and this induces net charges on the faces of the unit cell under the mechanical stress, which results in a net dipole moment. The sum of these dipole moments from all the unit cells leads to charge separation and generates electrical polarization in piezoelectric materials (Figure 2b). The most important thing in this system is that the materials must not have a center of symmetry, because the sum of net dipole moments is zero if the materials have a symmetry center.

Fortunately, M13 phages have structural properties that can make them suitable for piezoelectric properties. M13 phages are composed of ssDNA and capsid proteins, and each capsid protein has a dipole moment because these capsid proteins are made up of three parts—a positively charged area, a neutral charged area, and a negatively charged area. Especially, 2700 pVIII major coat proteins, which are the body-coat proteins, are assembled on the ssDNA with a 20° tilt angle with respect to the phage long axis and are arranged in right-handed helical structures. These major coat proteins form a pentagonal structure, which means that M13 phages have five-fold rotational symmetry, two-fold screw symmetry, and no inversion center (Figure 2c) [46,47]. Therefore, M13 phages can have piezoelectric properties. Interestingly, these complex structural characteristics of M13 phages allow us to use various piezoelectric properties. When stress is applied along the phage long axis, net dipole moments and electrical polarization are generated along the direction of applied stress (Figure 2d). On the other hand, when the stress is applied along the body (phage short axis), net dipole moments and electrical polarization are generated in two different directions (Figure 2e,f). Due to these characteristics, various types of piezoelectric energy harvesting devices can be developed.

## 4. Surface Modification of M13 Bacteriophages through Genetic Engineering

One of the great advantages of M13 phages as functional materials is the possibility of surface modification through genetic engineering. The most frequently used and well-established method to modify the genes of phages is recombinant DNA technology, which involves the insertion of foreign genes into the bacterial plasmids. Especially, M13mp phage vectors are usually used for engineering M13 phages. By incorporating foreign DNA, converting certain DNA into foreign DNA, and deleting specific DNA with enzymes, a high density of functional peptides and proteins can be simultaneously displayed on the M13 phage’s coat proteins [45]. This technique enables us to design the surface molecular structures of M13 phages according to their purpose. For example, J.-W. Oh et al. developed the highly trinitrotoluene (TNT)-selective sensors based on phage colorimetric structures by expressing the AXXXWHWQXXDP (WHW) peptide sequence (which shows excellent binding affinity to TNT molecules) on pVIII major coat proteins [48]. J. Wang et al. reported that RGD phages induce osteogenesis and angiogenesis by activating the endothelialization and osteogenic differentiation of mesenchymal stem cells. In this work, RGD and RGD/PHSRN (combination of RGD and PHSRN) peptides, which interact with integrin, have a key role in adhesion with fibronectin [49,50].

**Figure 3:**
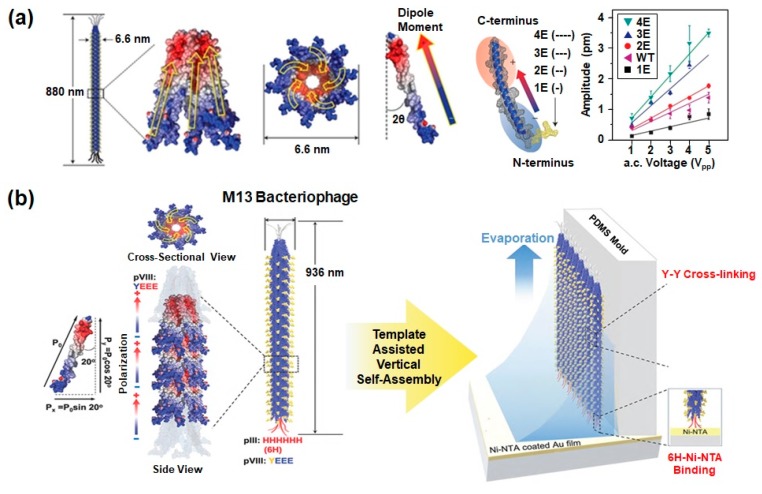
Surface modification of M13 bacteriophages through genetic engineering. (**a**) AEGDP (1E), AEEGDP (2E), AEEEGDP (3E), and AEEEEDP (4E) peptide sequences were expressed on the outer surfaces of the pVIII major coat protein of phages to enhance the piezoelectric properties. The M13 phage is 880 nm in length and 6.6 nm in diameter, which is covered by 2700 pVIII coat proteins and has five copies each of pIII and pIX proteins. The dipole moment is directed from the N-terminus (blue) to the C-terminus (red). Reproduced with permission from [28]. Copyright Nature Research, 2012. (**b**) HHHHHH peptide sequence expressed at the end of the N-terminus of the pIII minor coat protein for specific binding of phages on the Ni-NTA surface. The YEEE peptide motif is expressed between the first and fourth residues at the N-terminal of the pVIII major coat protein to enhance the mechanical stability by Y-Y cross-linkage. Reproduced with permission from [29]. Copyright American Chemical Society, 2019.

These genetic modifications are also very useful in fabricating energy harvesting devices. Most energy harvesting devices (e.g., piezoelectric and triboelectric devices) have a direct correlation to surface charges and dipole moments. Therefore, the number of charges on the outer surfaces of M13 phages should be increased to improve the power of energy generators. For this purpose, B. Y. Lee et al. expressed AEGDP (1E), AEEGDP (2E), AEEEGDP (3E), and AEEEEDP (4E) peptide sequences on the outer surfaces of the pVIII major coat protein of M13 phages (Figure 3a) [28]. In the case of vertically aligned phages, the HHHHHH peptide sequence was expressed at the N-terminus of the pIII minor coat protein with a spacer GGGS as a specific binder with Ni-NTA surface (Figure 3b). The YEEE peptide was also expressed between the first and fourth residues at the N-terminal of the pVIII major coat protein for enhancing mechanical stability by Y-Y cross-linkage between phages through UV illumination [29].

Likewise, we can improve the physical and chemical properties and extend the range of applications through genetic molecular design. In the future, we expect that chemical modification (e.g., bioconjugate techniques and cross-linking) as well as genetic modification will be used for the further improvement of physical properties.

## 5. Piezoelectric Properties of M13 Bacteriophages

**Figure 4:**
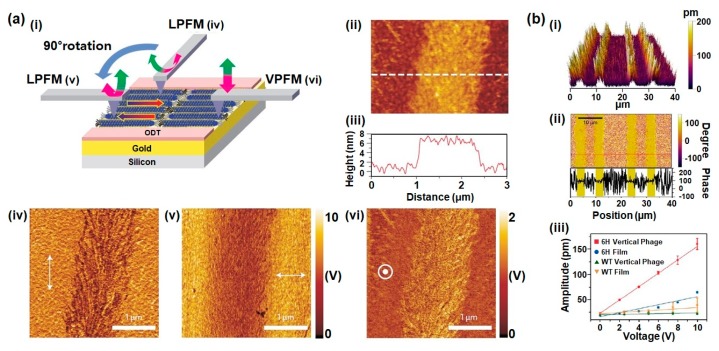
Piezoelectric properties of M13 bacteriophages. (**a**) Schematic of piezoresponse force microscopy (PFM) measurement (i); AFM topography (ii); height profile (iii); lateral PFM image along the phage long axis direction (iv); lateral PFM image obtained after changing the scanning direction by 90° (v); and vertical PFM image (vi) of the phage monolayer film. The resulting effective piezoelectric coefficients of 1E, 2E, 3E, and 4E phages were 0.14 ± 0.03 pm V^−1^, 0.35 ± 0.03 pm V^−1^, 0.55 ± 0.03 pm V^−1^, and 0.70 ± 0.05 pm V^−1^, respectively. Reproduced with permission from [28]. Copyright Nature Research, 2012. (**b**) PFM image (i), PFM phase image (ii) of vertically aligned phages which exhibits unidirectional polarization in the out-of-plane direction, and comparison of out-of-plane PFM amplitude versus applied voltage along the aligned direction (iii). The resulting effective piezoelectric coefficients (*d_eff_*) of 6H vertical phage, 6H film, WT vertical phage, and WT film were ~13.2 pm V^−1^, ~3.96 pm V^−1^, ~0.35 pm V^−1^ and 1.22 pm V^−1^, respectively. Reproduced with permission from [29]. Copyright American Chemical Society, 2019.

In general, the M13 phage is covered with 2700 pVIII coat major proteins, and the individual coat protein of phages is roughly divided into three sections: a positively charged region (C-terminus), a neutral region, and a negatively charged region (N-terminus). Owing to this adequate arrangement of charges, each coat protein has a dipole moment which is directed from the N-terminus to the C-terminus. Furthermore, the positively charged region of coat proteins is bound to central single-stranded DNA with a 20° tilt angle with respect to the phage long axis and α-helical structure when they are released from the host cell. The resulting structures of assembled pVIII coat proteins have five-fold rotational symmetry, two-fold screw symmetry, and no inversion center. Based on this fundamental study of phage structures, we can easily predict that M13 phages can present strong piezoelectric properties due to their permanent axial polarization caused by the net dipole moment in the pVIII proteins [28]. In 2012, B.Y. Lee et al. successfully observed the piezoelectric properties of M13 phages by using piezoresponse force microscopy (PFM) (Figure 4a). For this study, they prepared the phage monolayer sample by vertically pulling an octadecanethiol (ODT)/cysteamine patterned substrate from the phage suspension at a constant speed. Then, the piezoelectric properties of wild-type, 1E, 2E, 3E and 4E phages were measured by PFM. Vertical PFM measurements revealed that the effective piezoelectric coefficients (*d_eff_*) of the wild-type phage was 0.30 ± 0.03 pm V^−1^. The coefficients of 1E, 2E, 3E, and 4E phages were 0.14 ± 0.03 pm V^−1^, 0.35 ± 0.03 pm V^−1^, 0.55 ± 0.03 pm V^−1^, and 0.70 ± 0.05 pm V^−1^, respectively. This indicates that the coefficients improve as the surface charges of phages increase [28]. To further enhance the piezoelectric properties, they fabricated a multilayer phage film with 100 nm thickness. The multilayer film exhibited an increased effective piezoelectric coefficient (3.9 ± 0.05 pm V^−1^). Although this value is lower than the *d_33_* values of periodically-poled lithium niobate (PPLN) (13.2 pm V^−1^), it is higher than collagen (1.1 pm V^−1^) and other natural biomaterials. In addition, the effective piezoelectric coefficient of M13 phage films is further enhanced by fabricating vertically aligned phage nanostructures. These vertically assembled phages exhibited unidirectionally oriented piezoelectric polarization with an effective vertical piezoelectric coefficient of 13.2 pm V^−1^ (Figure 4b). Therefore, M13 phages are the best natural biomaterials for developing piezoelectric energy generators based on biomaterials [28].

## 6. Developments and Applications of M13 Bacteriophage Based Piezoelectric Energy Harvesting Devices

**Figure 5:**
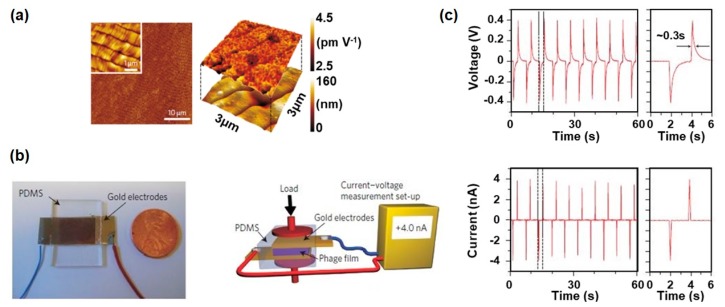
Characterization of an M13-bacteriophage-based piezoelectric energy generator (drop-casted film). (**a**) AFM topography image of phage film prepared by drop-casting, showing the long-range ordered smectic-phase liquid-crystalline film structure. Reproduced with permission from [28]. Copyright Nature Research, 2012. (**b**) Photograph of an M13 phage-based piezoelectric energy generator and schematic of piezoelectric energy generation measurement set-up. Reproduced with permission from [28]. Copyright Nature Research, 2012. (**c**) Open-circuit voltage and short-circuit current signal from the M13 phage-based piezoelectric energy generator. The device produced a current of 6 nA and a voltage of 400 mV when they use 4E phages. Reproduced with permission from [28]. Copyright Nature Research, 2012.

Due to their excellent piezoelectric properties, the group of Prof. Lee at UC Berkeley first fabricated M13 phage-based piezoelectric energy generators in 2012 [28]. They prepared well-ordered self-assembled multilayer films based on M13 phages onto gold-coated flexible substrates by using a drop and evaporation method (Figure 5a). During the evaporation process, M13 phages were self-assembled and formed long-range ordered smectic-phase liquid-crystalline films by their chiral and monodisperse characteristics. When they overlaid a counter gold-coated flexible substrate on the film and embedded the device between two 2.5-mm-thick polydimethylsiloxane (PDMS) matrices, they could fabricate phage-based energy generators (Figure 5b). The generating power of the device can be modulated by the surface modification of M13 phages through genetic engineering, and the device produced a current of 6 nA and a voltage of 400 mV when they use 4E phages (Figure 5c). This was a sufficient energy output to turn on a liquid-crystal display [28].

**Figure 6:**
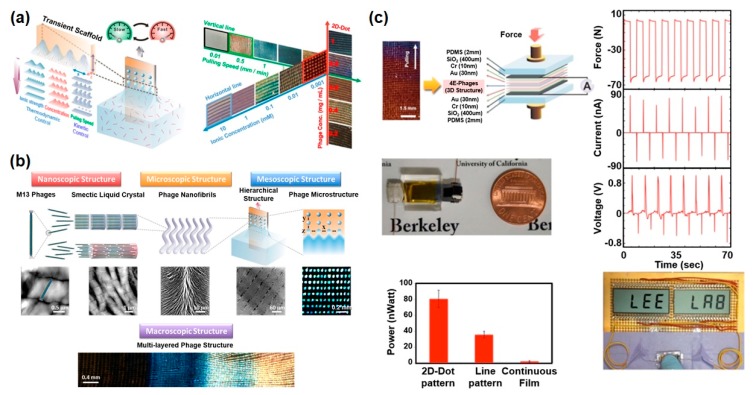
Characterization of an M13 bacteriophage-based piezoelectric energy generator. (biomimetic hierarchical structures). (**a**) Schematic illustration of the biomimetic hierarchical assembly process and structural transition diagram of phage films depending on phage concentration, ionic concentration, and pulling speed. Reproduced with permission from [30]. Copyright Elsevier, 2019. (**b**) Hierarchical structures of the phage-based films from nanoscale to macroscale. (single phage–phage filaments–fiber bundles–mesoscale periodic structure–macroscale band). (**c**) Piezoelectric characterization depending on phage structures. The 2D-dot patterns showed the highest piezoelectric performance, exhibiting peak values of 94 nA current and 0.95 V voltage. Reproduced with permission from [30]. Copyright Elsevier, 2019.

One of effective strategy for enhancing the power of phage-based energy generators is modulating the film morphology. Recently, K. Heo et al. reported a novel biomimetic assembly method for fabricating phage-based hierarchical structures with diverse surface morphologies by mimicking nature’s self-assembly system [30]. They modulated the meniscus by controlling the thermodynamic and kinetic parameters (i.e., phage concentration, ionic concentration, phage surface charge, and pulling speed) and created a hierarchically organized phage film with diverse morphology in a controlled manner as the meniscus can serve as a transient scaffold to guide phage self-assembly (Figure 6a). In this process, the shape of the meniscus can be systematically modulated due to a combination of multiple factors, such as fingering instability, Rayleigh instability, and elastocapillary instability. All of the resulting phage structures were long-range-ordered chiral phage films showing multiple levels of hierarchical organization from nano- to macro-scale (single phage–phage filaments–fiber bundles–mesoscale periodic structure–macroscale band) (Figure 6b) [30]. When they fabricated piezoelectric energy generators based on these hierarchically organized phage films, the device power was improved compared to previous drop-casted phage films. The continuous and line film patterns exhibited 6.3 and 56 nA short-circuit current and 0.36 and 0.75 V open-circuit voltage, respectively (Figure 6c). The 2D-dot patterns showed the highest piezoelectric performance, which exhibited peak values of 94 nA current and 0.95 V voltage. They claimed that these enhanced piezoelectric properties of 2D-dot phage patterns were mainly due to the enhanced crystallinity of the phage nanofilaments that were periodically organized in an active array, in contrast to other films. The enhanced power was available to display the words “VIRUS” and “LEE LAB” on a liquid-crystal display.

**Figure 7:**
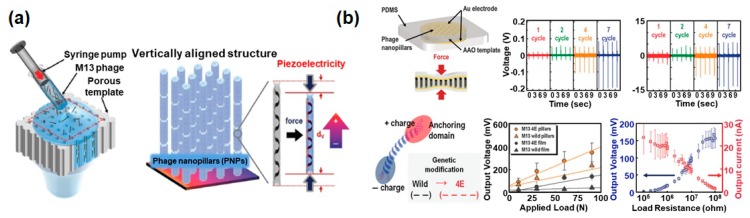
Characterization of an M13 bacteriophage-based piezoelectric energy generator (vertically aligned phage nanopillars). (**a**) Schematic showing the fabrication process and the resulting structures of vertically aligned M13 bacteriophage nanopillars. Reproduced with permission from [31]. Copyright The Royal Society of Chemistry, 2015. (**b**) Piezoelectric characterization of the phage nanopillar (PNP)-based energy generators. The 4E PNP-based energy generator produced 232 mV open−circuit voltage and 11.1 nA short-circuit current. Reproduced with permission from [31]. Copyright The Royal Society of Chemistry, 2015. AAO: anodic aluminium oxide.

Another strategy for improving the piezoelectric power is to change the direction of the mechanical force applied to the phage. As mentioned in Section 3, if we consider the direction of the dipole moment in the individual M13 phages, it is predicted that the accumulated charges are maximized when the mechanical force is applied in the vertical direction rather than the lateral direction (Figure 2d). However, we could not carry out the related research because of the absence of an effective process to vertically align the phages. Recently, D.-M. Shin et al. developed a robust and facile method to prepare vertically aligned the phages for the first time (Figure 7a) [31]. They extruded phage suspension into a porous anodic aluminium oxide (AAO) template at precisely controlled speeds and repeated this process until all holes of the porous template were completely filled with phages, resulting in the formation of phage nanopillars (PNPs). During this process, negatively charged 4E phages were randomly adsorbed on the positively charged inner surface of the porous template and spontaneously accumulated inside the pores due to their liquid-crystalline characteristics. Afterwards, they deposited bottom and top Au electrodes on the AAO template including the PNPs and encapsulated the whole device using PDMS to improve their stability. The 4E PNP-based energy generator produced a 232 mV open-circuit voltage and 1.1 nA short-circuit current (Figure 7b) [31]. The relatively low power of this device compared to what was expected is presumed to be a result of the difficulty of forming well-ordered liquid crystalline phage structures, and the direction of the dipole moments of individual phages is therefore randomly oriented.

**Figure 8:**
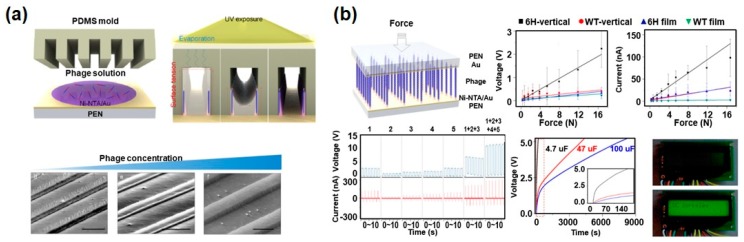
Characterization of an M13 bacteriophage-based piezoelectric energy generator (vertically aligned and unipolarized phage structures). (**a**) Schematic diagram of the fabrication process and scanning electron microscope (SEM) images of vertically aligned phages. Reproduced with permission from [29]. Copyright American Chemical Society, 2019. (**b**) Characterization of vertically aligned phage-based piezoelectric energy harvesters. This device exhibited a peak voltage of 2.8 V and a current of 120 nA. This is the largest power among phage-based energy generators. Reproduced with permission from [29]. Copyright American Chemical Society, 2019.

To overcome the limitation of the dipole alignment at the vertically oriented phage structures, J.H. Lee et al. developed a powerful method by combining the self-assembly of phages in a micro-fluidic channel and the surface modification of phages through genetic engineering (Figure 8a) [29]. To align the phages in the vertical direction, they tried to use a PDMS mold with micro-channels. When they dropped the phage suspension on the substrate and put on the PDMS mold, phage suspensions were infiltrated inside the micro-channels. The phages were vertically assembled on the wall of the PDMS micro-channels and cross-linked with each other as the solvent was evaporated with UV light exposure. The morphology and filling density of vertical phage structure could be controlled by the initial phage concentration. Furthermore, they controlled the direction of the dipole moment of the phages by changing the peptide sequence of minor coat proteins at the same time. Because they inserted hexa-histidine (6H) at the N-terminal of the minor coat protein (pIII) of the phages through genetic modification, the pIII proteins of all phages were strongly specifically bound with the nickel-nitrilotriacetic acid (Ni-NTA) modified substrate, which polarized the dipole moment of the M13 phages. Finally, they fabricated piezoelectric energy harvesters using the resulting vertically aligned and unipolarized phages, and the peak voltage reached 2.8 V, with a current of 120 nA (Figure 8b). This is the largest power among phage-based energy generators. Five integrated energy generators demonstrated the operation of a liquid-crystal display reading “UC Berkeley” [29].

Although the power of phage-based energy generators is improving with diverse strategies, the power of the devices is still too low. For practical applications of these devices, many ways for enhancing electrical properties and developing mass-production methods should be contrived.

Because these methods are to prepare the M13 phage film by self-assembly, the performance of the devices may be reduced when they are used for a long time. Fortunately, laterally assembled structures are very stable and robust, allowing the devices to run reliably for a long time [28,30]. However, vertically aligned structures are likely to be vulnerable to long-term use. This can be solved by using chemically cross-linked M13 phages [29] and filling rubbery buffer materials in the empty space.

As this technique is at an early stage of research, studies of the devices’ ideal operating conditions and toxicity issues have not been adequately carried out. However, by inferring from previous research based on M13 phages, the characteristics of these device can be predicted. Because the M13 phage is a biological material, optimized conditions for operating these devices will be room temperature (30 °C–70 °C) and low humidity [28]. However, these conditions can be modulated by surface modification. Through genetic engineering, we can modify the surface peptide motif to increase the hydrophobicity and cross-link phages to each other. Further, the M13 phage is known to be benign to humans because its host is *Escherichia coli* bacteria, not human cells [51,52,53]. Removing the infection motif in the pIII protein through genetic modification is also expected to be a good way to block the toxicity issues. Nevertheless, the study of M13 phages’ toxicity should be conducted in the near future.

Since these technologies are still in their early stages of research, it is too early to discuss mass-production for practical applications. Most of the techniques discussed here are not suitable for mass production, because they use new process methods rather than conventional fabrication techniques. However, because these novel fabrication processes are very simple and facile, there is a strong possibility of mass-production and scale-up in the future. Although one of the main issues for scale-up is mass-production of the M13 phages, we can solve this problem using huge fermenters in the factories, like with biosimilar drug and alcohol manufacturing. Although the manufacturing cost of these devices is more expensive than existing devices, the M13 phage-based devices have several strong advantages which are very important in the biomedical fields. The M13 phage has very high piezoelectric coefficient compared to other biomaterials and their surfaces can be easily modified by genetic engineering. Further, it is also possible to mass-produce them.

## 7. Conclusions and Future Perspective

Even though the piezoelectric properties of biomaterials are lower than other inorganic materials, it is very important to design novel piezoelectric biomaterials and develop functional devices because of their specific applications in biomedical field. In particular, M13 bacteriophages are very attractive materials due to their unique features which distinguish them from other materials, such as their similar structures with collagens, mass-amplification, genetic modification, liquid-crystalline phase transition, and excellent piezoelectric properties. Recently, taking advantage of these characteristics, many researchers have made a great deal of efforts to fabricate M13 phage-based piezoelectric energy harvesting devices. Among these devices, vertically aligned phage films exhibited the highest performance—a peak voltage of 2.8 V and a peak current of 120 nA [29].

However, it is still a challenge to develop high-performance piezoelectric energy generators based on M13 phages owing to the limitations of surface modification, structural, and dipole alignment control. Thus, the novel design of phage structures through genetic and chemical modification may improve the performance of devices. Further, fabricating triboelectric devices based on M13 phages will also be an effective way to enhance the power of devices.

Another strategy for enhancing the power of devices is to develop composite structures composed of organic and inorganic biomaterials. Recently, novel methods for coating inorganic materials on biomaterial surfaces are attracting the attention of many researchers because of their various applications in biomedical field. For example, some researchers have reported effective methods to coat the inorganic materials on M13 bacteriophage surfaces via biomineralization [54,55,56], while other researchers developed the strategies to cast metals on the surface of biological materials by using protein cage systems and self-assembly [57,58,59]. These methods are expected to be used to produce precursors for energy-harvesting devices and maximize the power of devices.

**Figure 9:**
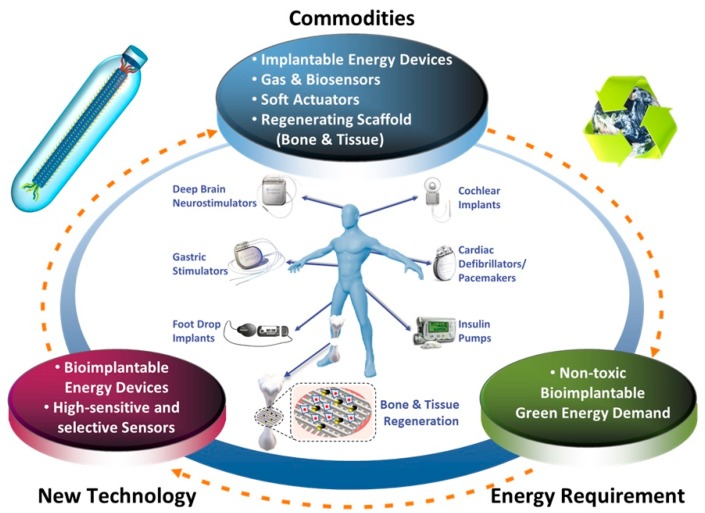
Schematic diagram showing the practical applications of M13 bacteriophage-based piezoelectric energy harvesting devices in the future.

High-performance energy harvesting devices based on biomaterials can be used in various fields, such as chemical/bio-sensors, artificial skin, bioimplantable energy devices, flexible electronics, soft robotics, and more (Figure 9). Especially, because there are many reports indicating that the surface charges and electrical signal can affect tissue regeneration, these piezoelectric biomaterials are also expected to be utilized for the development of biodegradable scaffolds for tissue engineering in the future.
